# Patient-Derived In Vitro Models for Drug Discovery in Colorectal Carcinoma

**DOI:** 10.3390/cancers12061423

**Published:** 2020-05-31

**Authors:** George M. Ramzy, Thibaud Koessler, Eloise Ducrey, Thomas McKee, Frédéric Ris, Nicolas Buchs, Laura Rubbia-Brandt, Pierre-Yves Dietrich, Patrycja Nowak-Sliwinska

**Affiliations:** 1 Molecular Pharmacology Group, School of Pharmaceutical Sciences, Institute of Pharmaceutical Sciences of Western Switzerland, University of Geneva, 1211 Geneva, Switzerland; George.Ramzy@unige.ch (G.M.R.); Eloise.Ducrey@unige.ch (E.D.); 2 Translational Research Center in Oncohaematology, University of Geneva, 1211 Geneva, Switzerland; 3 Department of Oncology, Geneva University Hospitals, 1211 Geneva, Switzerland; Thibaud.Kossler@hcuge.ch (T.K.); Pierre-Yves.Dietrich@unige.ch (P.-Y.D.); 4 Division of Clinical Pathology, Diagnostic Department, University Hospitals of Geneva (HUG), 1211 Geneva, Switzerland; Thomas.A.McKee@hcuge.ch (T.M.); Laura.Rubbia-Brandt@unige.ch (L.R.-B.); 5 Translational Department of Digestive and Transplant Surgery, Faculty of Medicine, Geneva University Hospitals, 1211 Geneva, Switzerland; Frederic.Ris@unige.ch (F.R.); Nicolas.Buchs@unige.ch (N.B.)

**Keywords:** colorectal cancer, organoids, 3D bioprinting, patient-derived xenograft, cancer-on-chip, drug combination

## Abstract

Lack of relevant preclinical models that reliably recapitulate the complexity and heterogeneity of human cancer has slowed down the development and approval of new anti-cancer therapies. Even though two-dimensional in vitro culture models remain widely used, they allow only partial cell-to-cell and cell-to-matrix interactions and therefore do not represent the complex nature of the tumor microenvironment. Therefore, better models reflecting intra-tumor heterogeneity need to be incorporated in the drug screening process to more reliably predict the efficacy of drug candidates. Classic methods of modelling colorectal carcinoma (CRC), while useful for many applications, carry numerous limitations. In this review, we address the recent advances in in vitro CRC model systems, ranging from conventional CRC patient-derived models, such as conditional reprogramming-based cell cultures, to more experimental and state-of-the-art models, such as cancer-on-chip platforms or liquid biopsy.

## 1. Introduction

Colorectal carcinoma (CRC) is the third most commonly diagnosed form of cancer in the world, with an estimated incidence of 1.8 million cases in 2018, and is expected to reach 2.2 million by 2030 [1,2]. Although cancer treatment in general has improved over the past few decades, the need for more personalized targeted therapies remains present, specifically for late-stage metastatic CRC (mCRC) patients for whom treatment options—and consequently overall survival rates—are limited [3].

The attrition rate of anticancer drugs candidates is very high, and only approximately 5% of drugs successfully complete phase III clinical trials [4,5]. One of the problems that might impair the development and approval of new anticancer therapies is the lack of relevant models that recapitulate the complexity of human cancer nature.

The main traits of an “ideal” CRC model for testing new treatment options reside in its capacity to resemble the in vivo conditions. This includes characteristics such as the genetic-, functional- and histological features of the patient’s tumor, along with sequential mutagenesis (i.e., loss of adenomatous polyposis coli, APC), followed by activating Kirsten rat sarcoma viral oncogene homolog (*KRAS*) mutations and loss of *TP53*, the presence of stromal- and immune cells, as well as the presence and composition of tumor stroma.

In patient-derived xenograft (PDX) models, small pieces of surgical patient tumor tissue are used for implantation into an immunodeficient mouse. Detailed protocols of the engraftment and propagation procedure for CRC PDX were described by several groups [6,7,8]. Different studies have demonstrated the potential use of PDX as a preclinical model in the drug screening cascade, as it can reliably predict and recapitulate CRC patient drug responses in colorectal cancer [9,10,11,12,13,14,15]. Although the tumors grow in a biologically more relevant microenvironment than can be provided in vitro, the mice are immunocompromised, therefore some of the complex interactions between the host and the tumor might not be preserved. Furthermore, some genetic and epigenetic changes may occur in the tumor cells during manipulations, such as resection, culture or implantation. Among several factors, the quality of the patient specimen, tumor type and stage, technique and time to implantation may affect the engraftment rates [16,17,18,19,20,21].

Multiple reports have shown that, after engraftment, the human tumor stroma is preserved, but is slowly replaced by murine stroma over time throughout the consequent passages [22]. In addition, in PDX models the microenvironment of the subcutaneous space differs greatly from that of the colon. The latter led scientists to develop orthotopic mouse models, where the tumor is directly implanted into the caecum. The main goal was to create an in vivo model that would allow tumor development locally in the colon, allowing all stages progression for CRC [23,24].

Genetically engineered mice (e.g., germline APC mutant models [25] or models presenting mismatch repair-deficiency [26]) and carcinogen-induced models [27] are widely used to investigate CRC and its treatment screenings. These models remain the most developed CRC in vivo models, due to their genetic controllability. Several of them are elegantly reviewed by others [28,29] and they are not discussed in this review.

Since the use of laboratory animals is laborious, time-consuming and expensive, in vitro models would greatly contribute to higher efficiency in drug screening. In addition, given that animal experimentation [30] is being widely discussed, the development of alternative in vitro models is needed to support the idea of reduction, refinement and replacement of laboratory animals. Even though drug discovery cannot be based purely on in vitro models, they can deliver important results that may, in turn, help in the reduction of further in vivo experiments.

**Figure 1:**
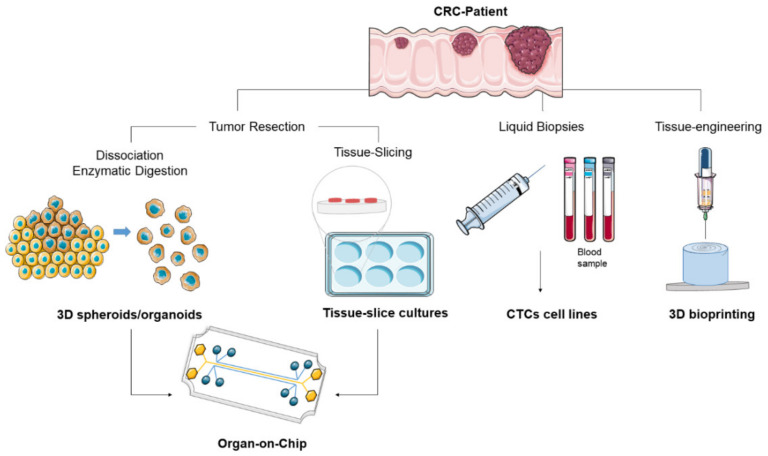
Overview of colorectal carcinoma (CRC) patient-derived preclinical models.

In this review we discuss a broad spectrum of in vitro CRC model systems, ranging from recent advances in conventional CRC patient-derived models, to more experimental and state-of-the-art technology models (Figure 1). We also suggest potentially attractive models used in other cancer types that would need further validation for CRC.

Recently developed CRC patient-derived models are usually established from freshly resected tumor tissue that undergoes enzymatic and mechanical digestion. Patient-derived models have emerged from liquid biopsies i.e., blood containing circulating tumor cells. Current advances in tissue engineering allowed patient-derived cells to be incorporated into a bioink and bioprinted into 3D constructs.

## 2. Conventional CRC In Vitro Models

### 2.1. Patient-Derived Tumor Cell Lines Cultivated in Two Dimensions

For decades, preclinical cancer research has been widely based on the use of cancer cell lines cultured in vitro and xenografts derived from these, grown in vivo. The culture of cells in vitro lead to the acquisition of multiple genetic and epigenetic alterations that diverge drastically from the original tumor they were derived from. Expanding and maintaining tissue-derived cell lines in culture often implicates the use of exogenous immortalization methods to keep cells in culture. These cells exhibit a similar gene expression and epigenetic profile, and can be propagated in vitro, into several germ layers, providing great potential for disease modelling such as cancer [31,32,33,34].

Recently, conditional reprogramming became widely used as a preclinical model in cancer research [35]. It is a co-culture based technology that makes it possible to efficiently expand patient-derived cells in culture medium supplemented with Rho kinase inhibitor (ROCK inhibitor, Y27632) and irradiated feeder fibroblasts [36]. Y27632 has been shown to induce the proliferative capacity of primary tumor cells resulting in efficient immortalization of cells into stratified epithelium without any DNA damage [37,38]. Several groups have elaborated specific protocols for cell isolation from various tumor types, including hepatocellular carcinoma [39], prostate cancer [40], tongue squamous cell carcinoma [41] and non-small lung cancers [42]. Liu et al. described a detailed protocol of CRC patient-derived cell cultures establishment from both cancerous and non-cancerous tissue biopsies that had the capacity to grow indefinitely in vitro, while maintaining phenotypic and genotypic features of the original tissue [36]. The study included freshly resected CRC tumors that generated approximately 10,000 cells after four weeks being in culture. This was done using conditional reprogramming, i.e., a novel next generation tool for long-term culture of primary epithelium cells derived from almost all origins without alteration of genetic background of primary cells. Moreover, Kodack et al. reported on a platform for functional testing on tumor-biopsy-derived cultures [43]. The criteria of a successful generation of colon cancer cell lines were defined as the point where the cells no longer required feeder cells to grow, could be cryopreserved and thawed and regrown, while maintaining the genotypic and phenotypic features. The authors also elaborated an immunofluorescence-based assay using a cocktail of monoclonal antibodies targeting cytokeratin (CK) 8 and cytokeratin 18 to specifically identify cancer cells, since both CK8/CK18 are nearly present in all tumors of epithelial origin.

The induction of conditional reprogramming in cancer cells is fast, and, unlike in the case of conventional cell lines, results in the generation of whole cell populations without clone selection. In addition, this technology makes it possible to maintain the phenotypic features of the primary tumor in culture. Future studies are still needed to confirm the genetic diversity within the isolated tumor cells.

In general, 2D cell cultures lack in vivo characteristics, such as tissue specific architecture, which can affect the proliferation of the cells and their response to external stimuli. This lack of complex cellular interactions fails to replicate the aggressiveness and heterogeneity of the disease, making 2D cultures poor models to predict drug response for complex diseases such as cancer. Two-dimensional models are used in a relatively high-throughput in vitro screening, but are constrained by the limited viability and the low and/or short proliferative capacity of the cultivated cells [20]. Moreover, the result highly depends on the isolation and culturing conditions, e.g., composition of the cell culture medium, seeding density and the addition of supplements or cellular matrixes [44,45,46,47].

Thus, traditional cell lines cultured in monolayers are not perfectly suited for complex CRC research and its further development led to creation of three-dimensional cell culture systems. To better recapitulate the organ and tumor complexity, researchers expanded technology of cell cultivation using the spheroid and organoid technology [46].

### 2.2. Patient-Derived Cells Cultivated in Three Dimensions

Spheroids are spherical cellular constructs, consisting of an external proliferating zone surrounding an internal quiescent zone [48]. The co-existence of these multilayers makes it possible to mimic the cellular heterogeneity observed in solid tumors [49,50]. We have recently developed a robust short-term 3D spheroid model, where human CRC cells were simultaneously co-cultured with human fibroblasts and human endothelial cells in a clinical relevant ratio [51].

Jeppesen et al. established short-term spheroids cultures obtained with a high success rate of over 80% from freshly-derived primary CRC tumors [52]. They show that the initial tumor fragment size does not affect the success rate of spheroid formation or the cellular characteristics of the spheroid, which preserve both the molecular and histological characteristics of CRC, while maintaining inter-patient sensitivity towards a given treatment.

The cell culture media composition has a major impact on the success rate of maintaining high viability of tumor-derived spheroids in culture. Available protocols remain inconsistent, as the report on various combinations of cell medium supplements and their positive effect on cell viability [47,53,54,55,56].

To date, the 3D CRC spheroids rarely contain immune cells [57,58]. Integrating a potential immune response in the patient-derived spheroid to a treatment might represent an important factor in the treatment design. Recently, CRC patient-derived spheroids were co-cultured with tumor infiltrating lymphocytes from the same patient [59] to study the infiltration, activation and function of immune cells in tumors. This study proved that both activated natural killer cells and activated T cells infiltrated the spheroids induced the death of cancer cells and disrupted the 3D spheroid structure. Heterotypic co-cultures of tumor spheroids with other immune cells types could further expand our knowledge of human anti-tumor immune responses.

Each of the above-mentioned models has its own advantages and drawbacks in terms of replicating the in vivo physiology of original tumor architecture, TME, cellular composition, as well as response to different exogenous stimuli. This is very often highly dependent on the initial patient specimen. These models are being constantly improved, to better recapitulate complex cancer biology. Further research regarding the automation, miniaturization and adaptation of spheroid co-culture models to human tumor types will make it possible to dynamically study the anti-tumor immune response. Several critical aspects of cell isolation and culture conditions need to be carefully considered when handling patient-derived in vitro material. The type of dissociation method (mechanical vs. enzymatic) used might influence the number and quality of isolated cells. In addition, the choice of an adequate antibiotic or a mixture of thereof, as well as the concentration of this in the culture medium, is critical to avoiding microbial contamination during transport and culture of the CRC cells [60]. Furthermore, evaluation of the purity and tumoral nature of the isolated cells, e.g., by flow cytometric analysis or immunohistochemistry, is essential for preserving a representative ratio of the different cell populations in the tumor of origin [60,61]. Patient-derived intestinal crypts (see Section 3.4) require the presence of Matrigel, and a defined mixture of the Lgr-5-ligand R-spondin, epidermal growth factors, and Noggin, that are known to be the most essential stem cell maintenance factors [62].

## 3. CRC Patient-Derived In Vitro Models

### 3.1. Patient-Derived Tissue Slice Culture

Three-dimensional culture systems have been developed to mimic in vivo tumors as closely as possible by considering two aspects of cancer: heterogeneity and stromal interactions. It is important to note that most of the models take only partially into account tumor complexity, and most of the components of the stroma are absent [63]. Patient-derived tissue slice culture model is a tumor slice of approximately 200–300 µm thick, which is enough to preserve histological features of the original tumor, as well as important cellular components such as immune, vascular and mesenchymal cells [64]. The latter are important key features of this culture model [65]. Patient-derived tissue slice culture models have already been established for various cancer types, such as prostate [66,67], breast [68] and lung [64]. Until now, only a few reports included this model to study CRC.

Sönnichsen et al. showed that slice culture from patient-derived colorectal tumor tissue represented similar morphological features to the original tumor over the observed cultivation period of 3 days. Persisting tumor cell proliferation in tissue slice culture under treatment with 5-FU, as highlighted in the study, can help identify a non-responding patients to a treatment, and therefore may help in preventing administration of ineffective treatment in clinically applicable timeframe [69]. Unlike 3D culture models, the initial step for this technique does not include an enzymatic digestion step of the tumor-tissue before the cells are stimulated to grow in 3D, which, in turn, makes it possible to maintain the complexity of the tumor without extra manipulation of the tissue [63]. Ironically, a key advantage of this model can also be a limiting factor, as the normalization of tumor cell fractions is a major inconvenience. The exact number of tumor cells in the tissue slice culture is not determined prior to the initiation step, which can greatly impact the reproducibility of the results [65]. While this model represents a promising technology to assess drug sensitivity in individual colorectal tumors, further correlations with clinical outcomes in larger cohorts of patients to validate the clinical application of the technique, are to be considered [69].

Tumor tissue slices of hepatic CRC metastases were used for the first time to evaluate the response to oxaliplatin, cetuximab and pembrolizumab and measure anti-proliferative and pro-apoptotic features of the tumor and morphometric changes [70]. Moreover, the RAS mutation status, as well as the immunohistochemical evaluation of microsatellite stability and checkpoint protein (PD1) expression, was assessed. This study identified non-responders and responding patients. Moreover, the original tumor sections showed moderate to high infiltrates of PD1 positive tumor-associated immune cells, indicating susceptibility to selected treatments.

One of the obstacles of the tissue-slice technique is the lack of long-term tissue preservation methods. In order to address this issue, Zhang et al. developed a new method of vitrification-based cryopreservation of tumor biopsies [71]. The patient-derived xenograft models were then successfully established. The observed drug responses in the xenograft model were consistent with those in tissue slice cultures performed in vitro. Importantly, the cultivation retained the heterogeneous architecture of the original tumor giving opportunity to further analysis of tumor biology.

### 3.2. Liquid Biopsy and Circulating Tumor Cells

Liquid biopsy refers to the analysis of biomarkers in any body fluid [72]. In oncology, liquid biopsy represents a non-invasive test using blood to analyze tumor-derived genetic materials (DNA, RNA and miRNA) and proteins that either can be circulating freely in the blood or incorporated in circulating tumor cells (CTCs) [73]. CTCs play an important key role in the understanding of the biology of metastasis in patients with cancer, as recent studies have shown that they are found in the blood of cancer patients, as single CTC or CTC clusters with a strong ability to seed metastasis [74]. CTCs are new potential biomarkers that have been recently employed as diagnostic, prognostic and predictive to a wide range of cancer type including breast, lung, prostate and colorectal cancers [75,76]. The detection and study of CTCs in peripheral blood have caught the attention of scientists over the past decade, mainly for their promising clinical implication. A recent study showed that the disruption of CTC clusters, which have been linked to high metastatic potential [77], increases the proportions of single CTCs in the blood stream, but suppresses overall metastasis formation, highlighting the importance of CTC clusters as potential therapeutic targets in cancer treatment [74].

The CellSearch^®^ platform, approved by the Food and Drug Administration (FDA), is currently used to identify, isolate and enumerate the CTC in the blood samples [78,79]. This technique consists mainly of the antibodies attached to magnetic beads against epithelial cell-adhesion molecule that are present on the surface of the CTC, and not on the healthy blood cells, separating magnetically the CTC from the bulk of other cells in the blood sample [80]. The low number of CTCs in peripheral blood makes it very challenging to establish their in vitro cultures. Recently, however, several groups managed to obtain CTC cell lines from patients with prostate [81] and breast cancer [82], two tumor types known to have a higher number of CTCs.

Cayrefourcq et al. reported for the first time the establishment of a permanent cell line from approximately 300 CTCs of one CRC patient, using the CellSearch^®^ platform [83]. This cell line has been cultured for more than one year. Thorough analysis of the cells at the genomic, transcriptomic, proteomic and secretomic levels showed high similarity to the tumor of the patient with colon cancer that they were derived from. This approach opened a new avenue for a potential platform for novel drug screening in CRC, by eventually generating single CTC or CTC spheres from patient-derived blood samples.

Another protocol that can be used to establish a CTC colorectal cancer patient-derived cell line was described by Grillet et al. The authors generated sufficient cellular material (5 million cells) within three weeks after sample collection, and then used it to perform cytotoxicity assays. The study offered preliminary clinical data suggesting that toxicity assays on CTC might predict patient response to drugs. A patient, from whom a CTC line was established, died after being treated with FOLFIRI (FOL = Folinic acid (Leucovorin) + F = 5-fluorouracil + IRI = irinotecan), a first line treatment in patients diagnosed with mCRC [84]. The CTC line was shown in this study to be resistant to this chemotherapy combination in vitro [85].

A potential future application of CTC in personalized medicine would be to develop CTC-derived organoids and CTC-derived xenografts that could be used in drug screening for CRC treatment, using minimally invasive methods, while reflecting to a high extent tumor heterogeneity ex vivo.

A major inconvenience of the use of liquid biopsy and CTC is the low concentration and yield of CTC extraction, especially in the blood of patients with CRC [83]. Moreover, major discrepancies have been highlighted, depending on the technique used for CTC detection, i.e., EpCAM antigen detection-based (CellSearch^®^) or cell size-based (ISET assay, based on the use of specific designed filtration membranes that allow size based exclusion of blood components, in different tumor types) [86]. The limited number of FDA approved technologies available for CTC detection and extraction makes the technology less accessible. Its wide application in clinical practice is also limited by its high costs. Lately, the development of microfluidic technology (see Section 3.3) is considered a potential alternative solution for CTC isolation [87]. Nevertheless, liquid biopsy holds great promise for revolutionizing cancer diagnostics in the future, to enable early detection.

### 3.3. Organ-on-Chip

The development of organ-on-chip (OOC) technology has made it possible to bridge the gap between conventional cell cultures, preclinical animal models and clinical trials in patients. In addition to recapitulating to a high extent the biology and physiology of the organ of origin, the OOC allow high-resolution-real-time imaging of living human cells in a functional human tissue and organ context [88].

OOC are microfluidic culture devices consisting essentially of flexible polymers, such as polydimethylsiloxane, containing perfused micro-channels harboring living cells that mimic in vivo organ architecture and physiology. The viability of cells can be maintained over an extended period (weeks to months) by flowing medium through the micro-channels. When the system is stabilized, medium can be replaced by whole blood perfusion for a couple of hours [89].

Multiple research groups managed to establish OOC platforms by replacing healthy cells and associated extracellular matrixes with those of cancers [90]. The concept behind the technology is to model cancer cell behavior within human-relevant tissue and organ microenvironments in vitro. OOC enable researchers to vary local cellular, molecular, chemical and biophysical parameters in a controlled manner, both individually and in precise combinations, while analyzing how they contribute to human cancer formation, progression and response to therapy. OOC have been developed for a wide range of solid tumors like brain [91], bladder [92], breast [93] and non-small lung cancer [94]. Not only has this technology been used to create organs and solid cancer-on-chip, but some research groups, like Zhou et al., have also employed it to isolate CTCs in cancer patients [87], or to model bone marrow angiogenesis [95]. Traditional static intestine models containing human epithelial cells (e.g., Cako-2 or HT-29 cell lines) cultured on extra-cellular matrix-coated membranes within the trans-well devices, could not support several intestine properties or its functions. Over the last few years, the intestine chips have been developed with increased complexity that include channels lined to human microvascular endothelium, immune cells or pathogenic bacteria, and allow interaction between them [96]. That, in turn, enabled studying physiology, as well as pathology of the intestine. A good example of such a device is the microfluidic two-channel gut chip, which contains human epithelial, endothelial, immune and microbial cells, co-existing on ECM-coated transparent silicon polymer [97]. In this model, the pneumatic application of suctions applied in cycles enabled device deformations, resembling the movements of intestine during the peristalsis. Importantly, under these conditions, epithelial cells that, in conventional 2D conditions, grow in monolayers, spontaneously undergo villus morphogenesis. This is an important improvement, as compared to organoids that do not experience physical stretching resulting from peristaltic contractions. Those villi are lined in a columnar manner similar to that in a real intestine [97]. Whether the human gut chip might be a potentially important application in CRC treatment remains to be demonstrated, but it is highly possible given its successful use in other complex disorders [96].

Along with an understanding of colonic epithelial cell behavior in the presence of microfabrication substrates, improved crypt isolation and 3D culture was an important step in the development of ‘organ-on-chip’ approaches for studies using primary colonic epithelium. Ahmad et al. reported on a protocol to standardize the isolation of intact murine colonic crypts by optimizing matrix concentrations on different surfaces i.e., PDMSs. The authors presented a reproducible low-cost crypt culture protocol, which may pave the way for further intestinal studies on patient-derived material using “organ-on-chip” platforms [98].

Concerning tumor-on-chip platforms, only a few studies are available, leaving great possibilities for further development. Carvalho et al. employed OOC technology to recapitulate the human colorectal tumor microenvironment, and assess the efficacy of gemcitabine loaded nanoparticles for the treatment of CRC using a microfluidic gradient [99]. In this device, the human CRC-like core, containing HCT-116 and HCoMECS cells, is surrounded by a vascularized microtissue, and serves as a tool to study radial drug penetration and efficacy of the microvascular network into a cancer-mimicking tissue. Although the oxygen gradient is not present in this device, its potential application is vast in CRC, or in solid tumors in general. This 3D microfluidic cell culture seems to be an extremely useful tool in the study of various phenomena, such as vascularization and oncogenesis under dynamic conditions. In the development of CRC, or its dissemination during the metastasis, the organ-on-chip-like microfluidic device has been developed [100]. This device, which merges microfluidics and photoconvertible protein technology, enables tracing the velocity of the circulating cells in the selectin-regulated process of adhesion and metastasis in a spatiotemporal manner.

Effectively, the OOC can be considered as a reliable platform to evaluate drug toxicity, given their high capacity to mimic in vivo like structures and functions. However, with these models, only the tissue or organ responses are considered without taking into account multi-organ interactions, which is crucial to evaluating the pharmacodynamics and the pharmacokinetics parameters of a drug [101]. To overcome this challenge, so called multiple-organs-on-chip (body-on-chip) were developed [102]. The latter technology recapitulates numerous organ interactions on a limited surface, while maintaining the highest degree of similarity to the in vivo situation. These systems usually do not require the use of pumps, using gravity to drive fluid flow to better replicate the physiologic flow. Oleaga et al. developed a system consisting of four different 2D tissue cultures (i.e., liver, cardiac, skeletal muscle and neuronal components), which were integrated within a single device to evaluate the toxicity of doxorubicin, valproic acid, acetaminophen and atorvastatin [103]. This phenotypic culture model exhibited a multi-organ toxicity response, representing the next generation of in vitro systems.

Esch et al. integrated liver and gastro-intestinal tract tissues within their device to evaluate intestinal barrier functions and metabolic rates of orally administered drugs and nutrients [104]. Fluidic flow through the organ chips was maintained via gravity and controlled passively via hydraulic resistances of the microfluidic channel network.

Another improvement of OOC was reported by Kassendra et al. on a generation of a “hybrid model” of OOC that integrated intestinal organoids, resulting in a higher similarity to the in vivo situation. They were able to recapitulate “normal” intestinal functions by integrating fluid flow and peristalsis-like motions, along with immune cells to a vascular compartment, which are all key factors to the normal intestinal physiology. In these culture conditions, biopsy-derived epithelial cells used were differentiated into villus-like epithelium (thin brush border of the colon epithelium). The primary human intestine chip model can be adapted for a wide range of applications, particularly in personalized medicine, by establishing a platform that could help investigate patient-specific disease mechanisms, as well as novel drug screening and anti-cancer therapy response [105].

### 3.4. Patient-Derived Organoids

Another recent development in human 3D in vitro technologies are the constructs derived from self-organizing stem cells that mimic the architecture, functionality and genetic feature of their corresponding organ [106]. The introduction of human patient-derived organoids (PDO) has enabled disease modelling, highlighting their great potential in biomedical applications, translational medicine and personalized therapy [107,108,109].

Moreover, PDO established from metastases taken by sequential biopsies at multiple time points, and multiple regions of heavily pre-treated CRC patients were used as pre-clinical models in co-clinical trials [110,111,112]. Those organoids were exposed to anti-cancer drugs, and the results were compared to patients’ responses in clinical trials. The findings revealed the capacity of PDO to mimic in vivo tumor organization, at histopathological, molecular and functional levels, and to predict patient’s treatment response [111].

Organoids can also be used to analyze mechanisms of drug resistance. Cancer stem cells expressing specific surface markers, such as CD44 and LGR5, known to be strongly associated with therapeutic drug resistance were co-cultured with epithelial and stromal cells to simulate the in vivo TME, with the use of an air-liquid interface (ALI) method [112,113]. ALI does not require exogenous growth factor supplementation and enables multilineage differentiation and sustained growth for over 60 days [112,114]. ALI patient-derived organoids presented higher resistance than CRC cell lines exposed to 5-fluorouracil and irinotecan (standard-of-care treatment of advanced CRC) [115].

Despite their advantages, PDO possess also shortcomings. Their self-organizing structure leads to different phenotypes between organoids, and might induce high background noise during drug-screening. The use of scaffolds and other bioengineering methods could help standardize cancer stem cell development into a specific and robust organoid morphology [116]. Lab-grown organoids showed some abnormalities, such as lack of cellular diversity and altered gene expression patterns [117]. Another important drawback of organoid development is time, as it takes several weeks to form a relevant organoid [111,118]. Moreover, the lack of some epithelial components, tumor stroma and microbiome remain major limitations of the PDO model [119]. Only very recent studies reported the incorporation of immune cells derived from CRC patient biopsies. The infiltration of immune cells was found to correlate with tumor growth and drug response. The ALI organoids could therefore be a promising approach to better understanding the tumor immune microenvironment and its impact on treatment response [120].

The PDO is an interesting in vitro model for preclinical drug development, due to its ability to mimic human physiopathology. As this still needs further technical and cost-effective improvements, it is unlikely that PDOs will fully replace existing drug development models.

### 3.5. Biomarkers-Based Drug Discovery

One other way to leverage tissue to predict drug sensitivity is to interrogate the tissue directly. For certain tumors, this approach has been a routine part of pathological assessment of patient samples. Breast cancers that express the receptor for estrogens and for progesterone are therefore rapidly and cheaply detected by immunohistochemistry, and can be treated effectively with one of the range of anti-estrogens available [121]. Similarly, B-cell lymphomas that express CD20 have had their prognosis transformed by the introduction of anti-CD20 therapeutic antibodies [122].

A recent extension of this approach has been an FDA-approved tumor treatment based on the tumor molecular characteristics, and not on the tissue origin or pathohistological type. This was based on the observation that patients, whose tumors have lost their mismatch repair machinery respond better to immunotherapy than patients with tumors, in which the machinery is intact [123]. The identification of this phenotype is routinely detected by immunohistochemistry. Other approaches, such as the evaluation of the immune response, tumor mutation burden and expression of programmed death-ligand (PD-L1) are also aimed at identifying patients whose tumors are likely to respond to immunotherapy [124].

With the democratization of molecular analysis of tumors many other anomalies have been and are being identified that can be targeted by specific therapies. The most established are the *EGFR* mutations in lung cancer [125] and *BRAF* mutations in melanoma with loss of the homology directed repair mechanism through loss of *BRCA1/2* or other associated genes being a more recent example [126]. However, at the moment, we are experiencing an explosion of new molecules that target different molecular abnormalities, a detailed review of which is beyond the scope of this review.

It is expected that, in the near future, we will witness a further expansion of our ability to characterize the phenotype of tumors, probably in the domain of expression analysis and proteomics through tissue analysis by mass cytometry [127]. These will allow the characterization of the pathways activated in different tumors, allowing the development of pathway instead of mutation directed therapies [128]. However, the high cost of the mass spectrometry remains the major constraint.

Moreover, Coppe et al. reported on a high-throughput kinase-activity mapping (HT-KAM) assay, which makes it possible to reveal the phosphor-catalytic signatures of tumors [129]. The HT-KAM is based on identifying catalytically hyper-active kinases in cell models or tissue, in order to highlight drug resistance and identify potential new drug targets. The authors synthesized a 228-peptide library that include 151 biological substrate protein regions phosphorylated by oncogenic kinases, and serve as combinatorial sensors of kinases phosphor-catalytic activity. Peptide phosphorylation signatures can be converted in kinase activity profiles, which will make it possible to identify the activity of druggable proto-oncogenic kinases in these models. This platform was tested to determine the new mechanism/targets of drug resistance in *BRAF^V600E^* CRC. In CRC cells (WiDr), which were exposed to treatment with a *BRAF* inhibitor vemurafenib [130], downregulation of the phospho-proteins MEK1/2 and ERK1/2 and upregulation in phospho-EGFR were observed, which was in line with the previously reported literature findings. The authors further investigated the kinase signatures and identified additional targets such as an increased activation of *AKT1*, *PDPK1* and *PRKCA* kinases. Synergy was observed when inhibitors of *AKT1*, *PDPk1* and *PRKCA* were paired with *BRAF^V600E^* targeting agents. This example of the screening platform introduces a new versatile approach of target-based drug discovery, eventually to be implemented alongside other strategies to improve precision medicine.

### 3.6. 3D Bioprinting

In the current unmet need of closer cellular and spatial complexity of a tumor in in vitro conditions, Boland et al. first deposed a patent for ink-jet printing of viable cells in 2006 [131]. During the last decade, tissue engineering has known significant advances with the emergence of 3D bioprinting. The latter showed potential to recreate tailored in vitro 3D heterocellular complex structures to replicate the heterogeneity of the native in vivo tissue [132]. High anatomic precise positioning of living cells embedded in a scaffold or scaffold-free based support, make it possible to obtain 3D structures [133]. A primordial component of the bioprinting procedure is the bioink. It consists of decellularized matrix, microcarriers, hydrogels and cells. Scaffold based approach consists in using bioink where cells are loaded in hydrogels (i.e., agarose, alginate, matrigel, etc.) that differ by their crosslinking properties and construct size they can create. Whereas, in scaffold-free models cell density is higher, cells self-organize and deposit an extracellular-matrix, which allows superior cell-cell interactions [134,135].

To date, there are no reports available on the use of 3D bioprinting to mimic intestine models. This could be probably explained by the complex intestine functions, containing absorption and secretion functions. Currently, only pharmacokinetics and toxicity studies have been reported using such technology with the use of CRC cell lines (i.e., Caco-2) [136]. Madden et al. established a 3D in vitro model based on 3D bilayered bioprinitng of human primary intestinal epithelium for the evaluation of pharmacokinetic parameters, i.e., absorption, distribution, metabolism and elimination). In this study, human intestinal epithelial cells (hIEC) were cultured for 21 days with human intestinal myofibroblast and printed on cell culture inserts allowing easy passage of compounds between apical and basolateral surfaces. Tissue architecture obtained with the 3D bioprinted model was compared to monolayer culture of Caco-2 cells, the gold standard cell line model of the intestinal barrier [137]. Cell-specific markers were identified such as CK19 (epithelial) or vimentin (myofibroblasts) and allowed to distinct both compartments; protein involved in tight junction (E-cadherin) and brush border formation (villus). Immunohistochemical staining for mucin-2 confirmed mucous secretion, which indicates normal intestinal function. Furthermore, genomic analysis of this 3D intestinal tissue showed that main intestinal phase I Cytochrome p450, which is the main family enzyme implicated in the metabolism of drugs and xenobiotics [138], especially CYP3A4, and phase II metabolic enzymes, as well as efflux transporters, were expressed at similar levels compared to the native intestine. The same enzymes in Caco-2 monolayers were upregulated, downregulated or undetectable. To confirm these results, CYP450 metabolism in 3D conditions was further evaluated using an inductor of CYP3A4 rifampicin. Its activity significantly induced the metabolization of its substrate Midazolam in the 3D intestine [136]. Those findings create a new venue for 3D bioprinting as preclinical model for evaluation and prediction of drug efficacy in drug development.

Langer et al. reported a study on patient-derived material integrated in the 3D bioprinting platform. Their approach was based on incorporating multiple cell types (fibroblasts, cancer cells including patient-derived cells or endothelial cells) into scaffold-free in vitro tissue of breast or pancreatic cancers. Various parameters including cell signaling, proliferation, and response to therapies were assessed. The 3D bioprinted model was used to create breast tumors using MCF-7 cell line. Ten-day-old tissues containing breast cancer cells were implanted into immunodeficient mice, and xenografts were treated using doxorubicin and targeted therapies (i.e., BEZ235, an mTOR inhibitor and sunitinib, a multi-target inhibitor). Sunitinib reduced significantly the vasculature density in the stromal compartment and increased collagen deposition.

The authors further created the 3D bioprinted pancreatic tumor model containing a PDX cell line surrounded by endothelial cells and pancreatic stellate cells (PSCs). The tumor tissue was treated for 6 days with gemcitabine (standard of care chemotherapy for pancreatic cancer), and results showed a dose-dependent response of cancer cell death. Furthermore, a patient-derived pancreatic tumor tissue, after enzymatic digestion, into the bioink surrounded by endothelial cells and PSCs. Bioprinted patient-derived cells maintained their tumorigenic properties, as confirmed by proliferation marker Ki67 staining, and showed similar morphological properties when compared to matching in vivo PDX or primary patient tumor [139].

Among the most common challenges faced by 3D bioprinting reside maintaining high cell viability and functionality, establishing constructs harboring in vivo like vascularization, obtaining resolution and reproducibility. Therefore, in order to improve cell viability during the bioprinting procedure, Colosi et al., employed a bioink that consists of a blend of alginate and gelatin methacroyl [140]. The authors optimized the formulation of a low viscosity bioink, which matches the physiological pH and osmolarity, and promotes cells adhesion as well as cellular migration, resulting in 80% of cell viability.

Furthermore, scientists from Rice University have recently developed a new bioprinting model that allows to create highly complex vascular networks, which recapitulate the body’s natural passageways for blood, air, lymph and other vital fluids. Grigoryan et al. underlined the fact that their bioprint of a tissue was not only phenotypically similar to its healthy counterpart in the organism but also able to “breath” like the organism, through the oxygenation and flow of red blood cells through a complex distensible vascular network model [141].

### 3.7. Clinical Point of View

The clinical management of CRC has not majorly changed over the last two decades, as compared to other tumor types. The need for more representative preclinical models in the drug screening cascade is essential, as the attrition rate for anti-cancer drugs is very high especially for CRC. Most therapeutic agents developed mainly target VEGF (bevacizumab, aflibercept) or its receptors (regorafenib) or EGFR (cetuximab, panitumab). These discoveries have been made using cell lines and xenografts [142].

**Figure 2:**
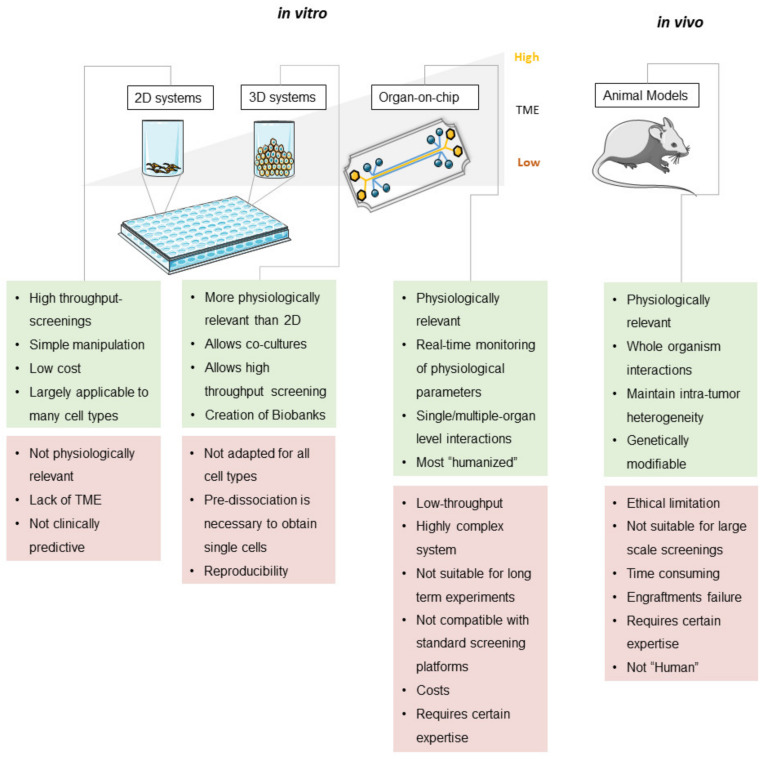
Advantages and weakness of CRC patient-derived models in preclinical studies.

Each of the presented platforms possesses its own strengths and drawbacks in terms of study design and expected outcome (see Figure 2). Patient-derived cell lines cultured in 2D monolayers are simple to manipulate, and usually allow for large high-throughput screenings. The lack of tumor microenvironment (TME) is improved in 3D organoids/spheroids, and they often retain the characteristics of the original tumor including tumor heterogeneity and complexity. In contrast to in vitro platforms, patient-derived xenograft models tumor microenvironment, but ethical limitations and host background represent main drawbacks of these models. Cancer-on-chip models have been recently developed as more physiologically relevant platforms [93,119,143,144].

Interestingly, in the case of the *BRAF^V600E^*, single or double inhibition (BRAF inhibitor and/or MEK inhibitor) has been unsuccessful in CRC treatment [130]. It was later shown that insensitivity to the double inhibition was due to a feedback activation of EGFR [145]. Current standard of care for *BRAF^V600E^*-mutated CRC involve the triple inhibition of BRAF, MEK and EGFR [146]. This remains a major consideration that needs to be integrated through patient-derived models for drug discovery in CRC. This said, it is fair to assume that the potential treatment or combination of treatment options have been missed, due to the model selection. The determinants of such paucity are the choice of the model and lack of integration into the model of tumor heterogeneity.

There are several expectations to be addressed from the clinician point of view regarding a patient-derived preclinical model in CRC. First, the treatment resulting from this process has to be more efficient than the current standard-of-care with similar or inferior toxicity. Second, when dealing with de novo CRC diagnosis or metastatic disease, two weeks is an acceptable turnaround time [147]. From a clinical point of view, from the diagnosis to the initial treatment, time should be as short as possible. However, preoperative workup and pre-habilitation are often time-consuming, but remain mandatory.

An adequate preoperative staging is important when considering neoadjuvant treatment. Microscopic tissue assessment of CRC by a pathologist aims at describing the complex composition and architecture of the tumor. The recent development of computer-aided approaches helped in advances also in this discipline. A machine learning-based approaches for automated analysis of digitized microscopic images of CRC samples with the goal to improve prognostic stratification of patients are already available [148]. For colon cancer, preoperative chemotherapy or even radio-chemotherapy showed interesting results with less surgical complications, but no impact on disease relapsing [149]. On the other hand, the assessment of the resected surgical specimen is the cornerstone before starting any adjuvant treatment. The importance to evaluate precisely the tumor, node, metastasis (TNM) stage is obvious, as it determines whether the patient should receive adjuvant treatment or not. Tissue availability is thus limited in localized disease, compared to the metastatic setting. A good collaboration between the pathologist and the researcher, dealing with the presented models, is fundamental, in order to maintain a high level of quality for the tumor staging. The part devoted for the research should not alter this quality.

It is important to underline that we are entering a new era of data-driven medicine. This is what offers the next-generation DNA sequencing (NGS). NGS describes the high-throughput technology that allows the sequencing of the entire human genome within a single day [150]. NGS-based diagnostic assays have achieved clinical utility, on one hand, by being a solid platform for direct therapeutic decision-making [151]. Today, the NGS enables to cluster patients’ tumor cases, based on their genomic profile, in virtual cohorts, in a way to determine whether the cancer of a specific patient is similar to that of another patient on the globe who received treatment A or B that saved them, matching genomic alterations with curated databases of evidence-based associations. Such platforms are being used in treatment of glioblastomas, lung, colorectal or gastrointestinal tumors, and can also be applied on liquid biopsy samples to help better diagnose, treat and monitor cancer in a less invasive manner.

Another important technological tool are the imaging techniques that allow 3D visualization of the patient-specific tumor phenotype with prognostic pre- and post-treatment relevance. New non-invasive imaging techniques, apart from structural evaluation, help in assessment of TME and certain hallmarks of cancer, as elegantly reviewed by García-Figueiras et al. [152].

Last but not least, the CRC in vitro model development might be supported by computer-aided approaches that facilitate experimental testing per se by guiding the researchers in the choice of tested conditions. It is extremely important especially in the context of CRC, where combinatory treatment approaches are mostly applied. Testing all combination options with multiple drugs is not trivial and requires a high time- and cost-effort. We have used the learning algorithm or statistical methods to lead experimental testing of multidrug combination candidates [153,154,155,156]. The generated data we modelled, made it possible to generate regression models that, in turn, enabled the elimination of ineffective and/or antagonistic compounds from the initial drug pool and led to identification of the most effective synergistic multidrug mixtures [155,156]. This approach brings the possibility of rapid patient-specific treatment optimization.

## 4. Conclusions

Despite the absence of an “ideal” preclinical model that would completely recapitulate the complexity of colorectal cancer with its stages and heterogeneity, as well as genomic signature, the choice of available models is wide-ranging. A careful decision on which model to employ should be taken, depending on a specific scientific question prior experimentation. A careful consideration of the advantages and shortcomings of each model, as presented above, should help in the most optimal model selection.

It is particularly important to mention that fundamental researchers should readily discuss the model choice with their clinical partners, i.e., oncologists, surgeons and pathologists, in order to secure the optimal conditions from tissue resection till its use in selected models. Already available in vitro models might provide very valuable information on several treatment aspects that can be further verified in more complex in vivo conditions. Model improvement should involve tumor phenotyping and genotyping (e.g., consensus molecular subtypes classifications), as well as a better representation of the TME [157]. Through the combination of multiple imaging along with biological and clinical information, the computer-aided-based platforms process the data using statistical models and the result is an accurate prediction of tumor growth and evolution. With the information gathered, eventual in vitro model can be further optimized through the characterization of a patient-specific tumor peculiarity.

## References

[1] Siegel R.L., A Torre L., Soerjomataram I., Hayes R.B., Bray F., Weber T.K., Jemal A. (2019). Global patterns and trends in colorectal cancer incidence in young adults. Gut.

[2] Bray F., Ferlay J., Soerjomataram I., Siegel R.L., Torre L.A., Jemal A. (2018). Global cancer statistics 2018: GLOBOCAN estimates of incidence and mortality worldwide for 36 cancers in 185 countries. CA Cancer J. Clin..

[3] McQuade R., Stojanovska V., De Leiris J., Nurgali K. (2017). Colorectal Cancer Chemotherapy: The Evolution of Treatment and New Approaches. Curr. Med. Chem..

[4] Centenera M.M., Raj G.V., Knudsen K.E., Tilley W.D., Butler L.M. (2013). Ex vivo culture of human prostate tissue and drug development. Nat. Rev. Urol..

[5] Hutchinson L., Kirk R. (2011). High drug attrition rates—Where are we going wrong?. Nat. Rev. Clin. Oncol..

[6] Uronis J.M., Osada T., McCall S.J., Yang X.Y., Mantyh C., Morse M.A., Lyerly H.K., Clary B.M., Hsu D.S. (2012). Histological and Molecular Evaluation of Patient-Derived Colorectal Cancer Explants. PLoS ONE.

[7] Burgenske D.M., Monsma D.J., MacKeigan J. (2018). Patient-Derived Xenograft Models of Colorectal Cancer: Procedures for Engraftment and Propagation. Colorectal Cancer.

[8] Ji X., Chen S., Guo Y., Li W., Qi X., Yang H., Xiao S., Fang G., Hu J., Wen C. (2017). Establishment and evaluation of four different types of patient-derived xenograft models. Cancer Cell Int..

[9] Bertotti A., Migliardi G., Galimi F., Sassi F., Torti D., Isella C., Corà D., Di Nicolantonio F., Buscarino M., Petti C. (2011). A Molecularly Annotated Platform of Patient-Derived Xenografts (“Xenopatients”) Identifies HER2 as an Effective Therapeutic Target in Cetuximab-Resistant Colorectal Cancer. Cancer Discov..

[10] Jung J., Kim J., Lim H.K., Kim K.M., Lee Y.S., Park J.S., Yoon D.-S. (2017). Establishing a colorectal cancer liver metastasis patient-derived tumor xenograft model for the evaluation of personalized chemotherapy. Ann. Surg. Treat. Res..

[11] Maletzki C., Bock S., Fruh P., Macius K., Witt A., Prall F., Linnebacher M. (2019). NSG mice as hosts for oncological precision medicine. Lab. Investig..

[12] Brown K.M., Xue A., Mittal A., Samra J.S., Smith R., Hugh T.J. (2016). Patient-derived xenograft models of colorectal cancer in pre-clinical research: A systematic review. Oncotarget.

[13] McIntyre R.E., Buczacki S.J., Arends M.J., Adams D.J. (2015). Mouse models of colorectal cancer as preclinical models. BioEssays.

[14] Gao H., Korn J.M., Ferretti S., Monahan J., Wang Y., Singh M., Zhang C., Schnell C., Yang G., Zhang Y. (2015). High-throughput screening using patient-derived tumor xenografts to predict clinical trial drug response. Nat. Med..

[15] Okada S., Vaeteewoottacharn K., Kariya R. (2019). Application of Highly Immunocompromised Mice for the Establishment of Patient-Derived Xenograft (PDX) Models. Cells.

[16] Katsiampoura A., Raghav K., Jiang Z.-Q., Menter D.G., Varkaris A., Morelli M.P., Manuel S., Wu J., Sorokin A.V., Rizi B.S. (2017). Modeling of Patient-Derived Xenografts in Colorectal Cancer. Mol. Cancer Ther..

[17] Rubio-Viqueira B., Jimeno A., Cusatis G., Zhang X., Iacobuzio-Donahue C., Karikari C., Shi C., Danenberg K., Danenberg P.V., Kuramochi H. (2006). An In vivo Platform for Translational Drug Development in Pancreatic Cancer. Clin. Cancer Res..

[18] Hidalgo M., Bruckheimer E., RajeshKumar N.V., Garrido-Laguna I., De Oliveira E., Rubio-Viqueira B., Strawn S., Wick M.J., Martell J., Sidransky D. (2011). A pilot clinical study of treatment guided by personalized tumorgrafts in patients with advanced cancer. Mol. Cancer Ther..

[19] Stebbing J., Paz K., Schwartz G.K., Wexler L.H., Maki R.G., Pollock R.E., Morris R., Cohen R., Shankar A., Blackman G. (2014). Patient-derived xenografts for individualized care in advanced sarcoma. Cancer.

[20] Fiore D., Di Giacomo F., Kyriakides P., Inghirami G. (2017). Patient-Derived-Tumor-Xenograft: Modeling cancer for basic and translational cancer research. Clin. Diagn. Pathol..

[21] Pauli C., Hopkins B.D., Prandi D., Shaw R., Fedrizzi T., Sboner A., Sailer V., Augello M., Puca L., Rosati R. (2017). Personalized In Vitro and In Vivo Cancer Models to Guide Precision Medicine. Cancer Discov..

[22] Chao C., Widen S.G., Wood T.G., Zatarain J.R., Johnson P., Gajjar A., Gomez G., Qiu S., Thompson J., Spratt H. (2017). Patient-Derived Xenografts from Colorectal Carcinoma: A Temporal and Hierarchical Study of Murine Stromal Cell Replacement. Anticancer. Res..

[23] O’Rourke K.P., Loizou E., Livshits G., Schatoff E.M., Baslan T., Manchado E., Simon J., Romesser P.B., Leach B., Han T. (2017). Transplantation of engineered organoids enables rapid generation of metastatic mouse models of colorectal cancer. Nat. Biotechnol..

[24] Céspedes M.V., Espina C., García-Cabezas M.A., Trias M., Boluda A., del Pulgar M.T.G., Sancho F.J., Nistal M., Lacal J.C., Mangues R. (2007). Orthotopic Microinjection of Human Colon Cancer Cells in Nude Mice Induces Tumor Foci in All Clinically Relevant Metastatic Sites. Am. J. Pathol..

[25] Swamy M.V., Patlolla J.M., Steele V.E., Kopelovich L., Reddy B.S., Rao C.V. (2006). Chemoprevention of Familial Adenomatous Polyposis by Low Doses of Atorvastatin and Celecoxib Given Individually and in Combination to APCMinMice. Cancer Res..

[26] Washington M.K., Powell A.E., Sullivan R., Sundberg J.P., A Wright N., Coffey R.J., Dove W. (2013). Pathology of rodent models of intestinal cancer: Progress report and recommendations. Gastroenterol..

[27] Chen J., Huang X.-F. (2009). The signal pathways in azoxymethane-induced colon cancer and preventive implications. Cancer Boil. Ther..

[28] Young M., Ordonez L., Clarke A. (2013). What are the best routes to effectively model human colorectal cancer?. Mol. Oncol..

[29] Golovko D., Kedrin D., Yilmaz O.H., Roper J. (2015). Colorectal cancer models for novel drug discovery. Expert Opin. Drug Discov..

[30] Tannenbaum J., Bennett B.T. (2015). Russell and Burch’s 3Rs then and now: The need for clarity in definition and purpose. J. Am. Assoc. Lab. Anim. Sci. JAALAS.

[31] Navarro A.M., Susanto E., Falk A., Wilhelm M. (2018). Modeling cancer using patient-derived induced pluripotent stem cells to understand development of childhood malignancies. Cell Death Discov..

[32] Curry E.L., Moad M., Robson C.N., Heer R. (2015). Using induced pluripotent stem cells as a tool for modelling carcinogenesis. World J. Stem Cells.

[33] Jesudoss M.X.D., Sachinidis A. (2019). Current Challenges of iPSC-Based Disease Modeling and Therapeutic Implications. Cells.

[34] Rowe R.G., Daley G.Q. (2019). Induced pluripotent stem cells in disease modelling and drug discovery. Nat. Rev. Genet..

[35] Suprynowicz F.A., Upadhyay G., Krawczyk E., Kramer S.C., Hebert J.D., Liu X., Yuan H., Cheluvaraju C., Clapp P., Boucher R.C. (2012). Conditionally reprogrammed cells represent a stem-like state of adult epithelial cells. Proc. Natl. Acad. Sci. USA.

[36] Liu X., Krawczyk E., A Suprynowicz F., Palechor-Ceron N., Yuan H., Dakic A., Simic V., Zheng Y.-L., Sripadhan P., Chen C. (2017). Conditional reprogramming and long-term expansion of normal and tumor cells from human biospecimens. Nat. Protoc..

[37] Chapman S., Liu X., Meyers C., Schlegel R., McBride A.A. (2010). Human keratinocytes are efficiently immortalized by a Rho kinase inhibitor. J. Clin. Investig..

[38] Terunuma A., Limgala R.P., Park C.J., Choudhary I., Vogel J.C. (2010). Efficient Procurement of Epithelial Stem Cells from Human Tissue Specimens Using a Rho-Associated Protein Kinase Inhibitor Y-27632. Tissue Eng. Part A.

[39] Wang Z., Bi B., Song H., Liu L., Zheng H., Wang S., Shen Z. (2019). Proliferation of human hepatocellular carcinoma cells from surgically resected specimens under conditionally reprogrammed culture. Mol. Med. Rep..

[40] Timofeeva O.A., Palechor-Ceron N., Li G., Yuan H., Krawczyk E., Zhong X., Liu G., Upadhyay G., Dakic A., Yu S. (2016). Conditionally reprogrammed normal and primary tumor prostate epithelial cells: A novel patient-derived cell model for studies of human prostate cancer. Oncotarget.

[41] Palechor-Ceron N., Krawczyk E., Dakic A., Simic V., Yuan H., Blancato J., Wang W., Hubbard F., Zheng Y.L., Dan H. (2019). Conditional Reprogramming for Patient-Derived Cancer Models and Next-Generation Living Biobanks. Cells.

[42] Correa B.R.S., Hu J., Penalva L.O.F., Schlegel R., Rimm D.L., Galante P.A.F., Agarwal S. (2018). Patient-derived conditionally reprogrammed cells maintain intra-tumor genetic heterogeneity. Sci. Rep..

[43] Kodack D.P., Farago A.F., Dastur A., Held M.A., Dardaei L., Friboulet L., Von Flotow F., Damon L.J., Lee D.-J., Parks M. (2017). Primary Patient-Derived Cancer Cells and Their Potential for Personalized Cancer Patient Care. Cell Rep..

[44] Dame M.K., Bhagavathula N., Mankey C., DaSilva M., Paruchuri T., Aslam M.N., Varani J. (2010). Human colon tissue in organ culture: Preservation of normal and neoplastic characteristics. Vitr. Cell. Dev. Boil. Anim..

[45] Zirvi K.A. (1991). Development of serum-free media for the growth of human gastrointestinal adenocarcinoma xenografts as primary tissue cultures. J. Cancer Res. Clin. Oncol..

[46] Sato T., Stange D.E., Ferrante M., Vries R.G., Van Es J.H., Brink S.V.D., Van Houdt W.J., Pronk A., Van Gorp J.M., Siersema P.D. (2011). Long-term Expansion of Epithelial Organoids from Human Colon, Adenoma, Adenocarcinoma, and Barrett’s Epithelium. Gastroenterology.

[47] Miyoshi H., Maekawa H., Kakizaki F., Yamaura T., Kawada K., Sakai Y., Taketo M.M. (2018). An improved method for culturing patient-derived colorectal cancer spheroids. Oncotarget.

[48] Edmondson R., Broglie J.J., Adcock A.F., Yang L. (2014). Three-Dimensional Cell Culture Systems and Their Applications in Drug Discovery and Cell-Based Biosensors. ASSAY Drug Dev. Technol..

[49] Zanoni M., Piccinini F., Arienti C., Zamagni A., Santi S., Polico R., Bevilacqua A., Tesei A. (2016). 3D tumor spheroid models for in vitro therapeutic screening: A systematic approach to enhance the biological relevance of data obtained. Sci. Rep..

[50] Cattin S., Ramont L., Ruegg C. (2018). Characterization and In Vivo Validation of a Three-Dimensional Multi-Cellular Culture Model to Study Heterotypic Interactions in Colorectal Cancer Cell Growth, Invasion and Metastasis. Front. Bioeng. Biotechnol..

[51] Zoetemelk M., Rausch M., Colin D.J., Dormond O., Nowak-Sliwinska P. (2019). Short-term 3D culture systems of various complexity for treatment optimization of colorectal carcinoma. Sci. Rep..

[52] Jeppesen M., Hagel G., Glenthoj A., Vainer B., Ibsen P., Harling H., Thastrup O., Jørgensen L.N., Thastrup J. (2017). Short-term spheroid culture of primary colorectal cancer cells as an in vitro model for personalizing cancer medicine. PLoS ONE.

[53] Weiswald L.-B., Richon S., Validire P., Briffod M., Lai-Kuen R., Cordelières F.P., Bertrand F., Dargere D., Massonnet G., Marangoni E. (2009). Newly characterised ex vivo colospheres as a three-dimensional colon cancer cell model of tumour aggressiveness. Br. J. Cancer.

[54] Ashley N., Jones M., Ouaret D., Wilding J., Bodmer W.F. (2014). Rapidly derived colorectal cancer cultures recapitulate parental cancer characteristics and enable personalized therapeutic assays. J. Pathol..

[55] Kondo J., Endo H., Okuyama H., Ishikawa O., Iishi H., Tsujii M., Ohue M., Inoue M. (2011). Retaining Cell-Cell Contact Enables Preparation and Culture of Spheroids Composed of Pure Primary Cancer Cells from Colorectal Cancer. Gastroenterology.

[56] Qureshi-Baig K., Ullmann P., Rodriguez F., Frasquilho S., Nazarov P.V., Haan S., Letellier E. (2016). What Do We Learn from Spheroid Culture Systems? Insights from Tumorspheres Derived from Primary Colon Cancer Tissue. PLoS ONE.

[57] Hoffmann O., Ilmberger C., Magosch S., Joka M., Jauch K.-W., Mayer B. (2015). Impact of the spheroid model complexity on drug response. J. Biotechnol..

[58] Hirt C., Papadimitropoulos A., Mele V., Muraro M.G., Mengus C., Iezzi G., Terracciano L., Martin I., Spagnoli G.C. (2014). “In vitro” 3D models of tumor-immune system interaction. Adv. Drug Deliv. Rev..

[59] Courau T., Bonnereau J., Chicoteau J., Bottois H., Remark R., Miranda L.A., Toubert A., Bléry M., Aparicio T., Allez M. (2019). Cocultures of human colorectal tumor spheroids with immune cells reveal the therapeutic potential of MICA/B and NKG2A targeting for cancer treatment. J. Immunother. Cancer.

[60] Failli A., Consolini R., Legitimo A., Spisni R., Castagna M., Romanini A., Crimaldi G., Miccoli P. (2009). The challenge of culturing human colorectal tumor cells: Establishment of a cell culture model by the comparison of different methodological approaches. Tumori J..

[61] Fan F., Bellister S., Lü J., Ye X., Boulbès D.R., Tozzi F., Sceusi E., Kopetz S., Tian F., Xia L. (2014). The requirement for freshly isolated human colorectal cancer (CRC) cells in isolating CRC stem cells. Br. J. Cancer.

[62] Pereira J.F.D.S., Awatade N.T., Loureiro C., Matos P., Amaral M.D., Jordan P. (2016). The third dimension: New developments in cell culture models for colorectal research. Cell. Mol. Life Sci..

[63] Meijer T.G., Naipal K.A., Jager A., Van Gent D.C. (2017). Ex vivotumor culture systems for functional drug testing and therapy response prediction. Future Sci. OA.

[64] Davies E.J., Dong M., Gutekunst M., Närhi K., Van Zoggel H.J.A.A., Blom S., Nagaraj A., Metsalu T., Oswald E., Erkens-Schulze S. (2015). Capturing complex tumour biology in vitro: Histological and molecular characterisation of precision cut slices. Sci. Rep..

[65] Brand D.V.D., Massuger L.F., Brock R., Verdurmen W.P. (2017). Mimicking Tumors: Toward More Predictive In Vitro Models for Peptide- and Protein-Conjugated Drugs. Bioconjug. Chem..

[66] Bläuer M., Tammela T.L., Ylikomi T. (2008). A novel tissue-slice culture model for non-malignant human prostate. Cell Tissue Res..

[67] Van De Merbel M., Van Der Horst G., Van Der Mark M.H., Van Uhm J.I.M., Van Gennep E.J., Kloen P., Beimers L., Pelger R.C.M., Van Der Pluijm G. (2018). An ex vivo Tissue Culture Model for the Assessment of Individualized Drug Responses in Prostate and Bladder Cancer. Front. Oncol..

[68] Carranza-Torres I.E., Guzmán-Delgado N.E., Coronado-Martínez C., Bañuelos-García J.I., Valdez E.V., Morán-Martínez J., Carranza-Rosales P. (2015). Organotypic Culture of Breast Tumor Explants as a Multicellular System for the Screening of Natural Compounds with Antineoplastic Potential. BioMed Res. Int..

[69] Sönnichsen R., Hennig L., Blaschke V., Winter K., Körfer J., Hähnel S., Monecke A., Wittekind C., Jansen-Winkeln B., Thieme R. (2018). Individual Susceptibility Analysis Using Patient-derived Slice Cultures of Colorectal Carcinoma. Clin. Color. Cancer.

[70] Martin S.Z., Wagner D.C., Hörner N., Horst D., Lang H., Tagscherer K.E., Roth W. (2019). Ex vivo tissue slice culture system to measure drug-response rates of hepatic metastatic colorectal cancer. BMC Cancer.

[71] Zhang Y., Huang W., Yang Q., Zhang H., Zhu X., Zeng M., Zhou X., Wang Z., Li W., Jing H. (2019). Cryopreserved biopsy tissues of rectal cancer liver metastasis for assessment of anticancer drug response in vitro and in vivo. Oncol. Rep..

[72] Siravegna G., Marsoni S., Siena S., Bardelli A. (2017). Integrating liquid biopsies into the management of cancer. Nat. Rev. Clin. Oncol..

[73] Normanno N., Cervantes A., Ciardiello F., De Luca A., Pinto C. (2018). The liquid biopsy in the management of colorectal cancer patients: Current applications and future scenarios. Cancer Treat. Rev..

[74] Gkountela S., Castro-Giner F., Szczerba B.M., Vetter M., Landin J., Scherrer R., Krol I., Scheidmann M.C., Beisel C., Stirnimann C.U. (2019). Circulating Tumor Cell Clustering Shapes DNA Methylation to Enable Metastasis Seeding. Cell.

[75] Cabel L., Proudhon C., Gortais H., Loirat D., Coussy F., Pierga J.-Y., Bidard F.-C. (2017). Circulating tumor cells: Clinical validity and utility. Int. J. Clin. Oncol..

[76] Bork U., Rahbari N.N., Schölch S., Reissfelder C., Kahlert C., Büchler M.W., Weitz J., Koch M. (2015). Circulating tumour cells and outcome in non-metastatic colorectal cancer: A prospective study. Br. J. Cancer.

[77] Aceto N., Bardia A., Miyamoto D.T., Donaldson M.C., Wittner B.S., Spencer J.A., Yu M., Pely A., Engstrom A., Zhu H. (2014). Circulating tumor cell clusters are oligoclonal precursors of breast cancer metastasis. Cell.

[78] Andree K.C., Van Dalum G., Terstappen L.W.M.M. (2015). Challenges in circulating tumor cell detection by the CellSearch system. Mol. Oncol..

[79] Wang L., Balasubramanian P., Chen A.P., Kummar S., Evrard Y.A., Kinders R.J. (2016). Promise and limits of the CellSearch platform for evaluating pharmacodynamics in circulating tumor cells. Semin. Oncol..

[80] Williams S.C.P. (2013). Circulating tumor cells. Proc. Natl. Acad. Sci. USA.

[81] Gao D., Vela I., Sboner A., Iaquinta P.J., Karthaus W.R., Gopalan A., Dowling C., Wanjala J.N., Undvall E.A., Arora V.K. (2014). Organoid cultures derived from patients with advanced prostate cancer. Cell.

[82] Zhang L., Ridgway L.D., Wetzel M.D., Ngo J., Yin W., Kumar D., Goodman J.C., Groves M.D., Marchetti D. (2013). The Identification and Characterization of Breast Cancer CTCs Competent for Brain Metastasis. Sci. Transl. Med..

[83] Cayrefourcq L., Mazard T., Joosse S., Solassol J., Ramos J., Assenat E., Schumacher U., Costes-Martineau V., Maudelonde T., Pantel K. (2015). Establishment and Characterization of a Cell Line from Human Circulating Colon Cancer Cells. Cancer Res..

[84] Souglakos J., Androulakis N., Syrigos K., Polyzos A., Ziras N., Athanasiadis A., Kakolyris S., Tsousis S., Kouroussis C., Vamvakas L. (2006). FOLFOXIRI (folinic acid, 5-fluorouracil, oxaliplatin and irinotecan) vs FOLFIRI (folinic acid, 5-fluorouracil and irinotecan) as first-line treatment in metastatic colorectal cancer (MCC): A multicentre randomised phase III trial from the Hellenic Oncology Research Group (HORG). Br. J. Cancer.

[85] Grillet F., Bayet E., Villeronce O., Zappia L., Lagerqvist E.L., Lunke S., Charafe-Jauffret E., Pham K., Molck C., Rolland N. (2016). Circulating tumour cells from patients with colorectal cancer have cancer stem cell hallmarks inex vivoculture. Gut.

[86] Farace F., Massard C., Vimond N., Drusch F., Jacques N., Billiot F., Laplanche A., Chauchereau A., Lacroix L., Planchard D. (2011). A direct comparison of CellSearch and ISET for circulating tumour-cell detection in patients with metastatic carcinomas. Br. J. Cancer.

[87] Zhou J., Kulasinghe A., Bogseth A., O’Byrne K., Punyadeera C., Papautsky I. (2019). Isolation of circulating tumor cells in non-small-cell-lung-cancer patients using a multi-flow microfluidic channel. Microsyst. Nanoeng..

[88] Esch E.W., Bahinski A., Huh D. (2015). Organs-on-chips at the frontiers of drug discovery. Nat. Rev. Drug Discov..

[89] Sontheimer-Phelps A., Hassell B.A., Ingber D.E. (2019). Modelling cancer in microfluidic human organs-on-chips. Nat. Rev. Cancer.

[90] Zhang Y.S., Zhang Y.-N., Zhang W. (2017). Cancer-on-a-chip systems at the frontier of nanomedicine. Drug Discov. Today.

[91] Akay M., Hite J., Avci N.G., Fan Y., Akay Y., Lu G., Zhu J.-J. (2018). Drug Screening of Human GBM Spheroids in Brain Cancer Chip. Sci. Rep..

[92] Liu P.-F., Cao Y.-W., Zhang S.-D., Zhao Y., Liu X.-G., Shi H.-Q., Hu K.-Y., Zhu G.-Q., Ma B., Niu H.-T. (2015). A bladder cancer microenvironment simulation system based on a microfluidic co-culture model. Oncotarget.

[93] Pradhan S., Smith A.M., Garson C.J., Hassani I., Seeto W.J., Pant K., Arnold R., Prabhakarpandian B., Lipke E. (2018). A Microvascularized Tumor-mimetic Platform for Assessing Anti-cancer Drug Efficacy. Sci. Rep..

[94] Yu T., Guo Z., Fan H., Song J., Liu Y., Gao Z., Wang Q. (2016). Cancer-associated fibroblasts promote non-small cell lung cancer cell invasion by upregulation of glucose-regulated protein 78 (GRP78) expression in an integrated bionic microfluidic device. Oncotarget.

[95] Zheng Y., Sun Y., Yu X., Shao Y., Zhang P., Dai G., Fu J. (2016). Angiogenesis in Liquid Tumors: An In Vitro Assay for Leukemic-Cell-Induced Bone Marrow Angiogenesis. Adv. Heal. Mater..

[96] Bein A., Shin W., Jalili-Firoozinezhad S., Park M.H., Sontheimer-Phelps A., Tovaglieri A., Chalkiadaki A., Kim H.J., Ingber D.E. (2018). Microfluidic Organ-on-a-Chip Models of Human Intestine. Cell. Mol. Gastroenterol. Hepatol..

[97] Kim H.J., Li H., Collins J.J., Ingber D.E. (2015). Contributions of microbiome and mechanical deformation to intestinal bacterial overgrowth and inflammation in a human gut-on-a-chip. Proc. Natl. Acad. Sci. USA.

[98] Ahmad A.A., Wang Y., Gracz A.D., Sims C.E., Magness S.T., Allbritton N.L. (2014). Optimization of 3-D organotypic primary colonic cultures for organ-on-chip applications. J. Boil. Eng..

[99] Carvalho M., Barata D., Teixeira L.S.M., Giselbrecht S., Reis R.L., Oliveira J.M., Truckenmüller R., Habibovic P. (2019). Colorectal tumor-on-a-chip system: A 3D tool for precision onco-nanomedicine. Sci. Adv..

[100] Edwards E.E., Birmingham K.G., O’Melia M.J., Oh J., Thomas S.N. (2018). Fluorometric Quantification of Single-Cell Velocities to Investigate Cancer Metastasis. Cell Syst..

[101] Zhao Y., Kankala R.K., Wang S.-B., Chen A.-Z. (2019). Multi-Organs-on-Chips: Towards Long-Term Biomedical Investigations. Molecules.

[102] Skardal A., Shupe T., Atala A. (2016). Organoid-on-a-chip and body-on-a-chip systems for drug screening and disease modeling. Drug Discov. Today.

[103] Oleaga C., Bernabini C., Smith A.S., Srinivasan B., Jackson M., McLamb W., Platt V., Bridges R., Cai Y., Santhanam N. (2016). Multi-Organ toxicity demonstration in a functional human in vitro system composed of four organs. Sci. Rep..

[104] Esch M.B., Ueno H., Applegate D.R., Shuler M.L. (2016). Modular, pumpless body-on-a-chip platform for the co-culture of GI tract epithelium and 3D primary liver tissue. Lab Chip.

[105] Kasendra M., Tovaglieri A., Sontheimer-Phelps A., Jalili-Firoozinezhad S., Bein A., Chalkiadaki A., Scholl W., Zhang C., Rickner H., Richmond C.A. (2018). Development of a primary human Small Intestine-on-a-Chip using biopsy-derived organoids. Sci. Rep..

[106] Lou Y.-R., Leung A. (2018). Next generation organoids for biomedical research and applications. Biotechnol. Adv..

[107] Dutta D., Heo I., Clevers H. (2017). Disease Modeling in Stem Cell-Derived 3D Organoid Systems. Trends Mol. Med..

[108] Drost J., Clevers H. (2018). Organoids in cancer research. Nat. Rev. Cancer.

[109] Van De Wetering M., Francies H.E., Francis J.M., Bounova G., Iorio F., Pronk A., Van Houdt W., Van Gorp J., Taylor-Weiner A., Kester L. (2015). Prospective derivation of a living organoid biobank of colorectal cancer patients. Cell.

[110] Nardella C., Lunardi A., Patnaik A., Cantley L.C., Pandolfi P.P. (2011). The APL Paradigm and the “Co-Clinical Trial” Project. Cancer Discov..

[111] Vlachogiannis G., Hedayat S., Vatsiou A., Jamin Y., Fernández-Mateos J., Khan K., Lampis A., Eason K., Huntingford I., Burke R. (2018). Patient-derived organoids model treatment response of metastatic gastrointestinal cancers. Science.

[112] Li X., Ootani A., Kuo C., Ivanov A.I. (2016). An Air–Liquid Interface Culture System for 3D Organoid Culture of Diverse Primary Gastrointestinal Tissues. Gastrointestinal Physiology and Diseases: Methods and Protocols.

[113] Katano T., Ootani A., Mizoshita T., Tanida S., Tsukamoto H., Ozeki K., Ebi M., Mori Y., Kataoka H., Kamiya T. (2013). Establishment of a long-term three-dimensional primary culture of mouse glandular stomach epithelial cells within the stem cell niche. Biochem. Biophys. Res. Commun..

[114] Li X., Nadauld L., Ootani A., Corney D.C., Pai R.K., Gevaert O., Cantrell M.A., Rack P.D., Neal J., Chan C.W.-M. (2014). Oncogenic transformation of diverse gastrointestinal tissues in primary organoid culture. Nat. Med..

[115] Elbadawy M., Usui T., Yamawaki H., Sasaki K. (2018). Development of an Experimental Model for Analyzing Drug Resistance in Colorectal Cancer. Cancers.

[116] Lancaster M.A., Corsini N.S., Wolfinger S., Gustafson E.H., Phillips A., Burkard T.R., Otani T., Livesey F.J., Knoblich J.A. (2017). Guided self-organization and cortical plate formation in human brain organoids. Nat. Biotechnol..

[117] Urbischek M., Rannikmae H., Foets T., Ravn K., Hyvönen M., De La Roche M. (2019). Organoid culture media formulated with growth factors of defined cellular activity. Sci. Rep..

[118] Lancaster M.A., Huch M. (2019). Disease modelling in human organoids. Dis. Model. Mech..

[119] Sasaki N., Clevers H. (2018). Studying cellular heterogeneity and drug sensitivity in colorectal cancer using organoid technology. Curr. Opin. Genet. Dev..

[120] Finnberg N.K., Gokare P., Lev A., Grivennikov S.I., Macfarlane A.W., Campbell K.S., Winters R.M., Kaputa K., Farma J.M., Abbas A.E.-S. (2017). Application of 3D tumoroid systems to define immune and cytotoxic therapeutic responses based on tumoroid and tissue slice culture molecular signatures. Oncotarget.

[121] Schettini F., Buono G., Cardalesi C., Desideri I., De Placido S., Del Mastro L. (2016). Hormone Receptor/Human Epidermal Growth Factor Receptor 2-positive breast cancer: Where we are now and where we are going?. Cancer Treat. Rev..

[122] Salles G., Barrett M., Foà R., Maurer J., O’Brien S., Valente N., Wenger M., Maloney D.G. (2017). Rituximab in B-Cell Hematologic Malignancies: A Review of 20 Years of Clinical Experience. Adv. Ther..

[123] Ciombor K.K., Goldberg R. (2018). Hypermutated Tumors and Immune Checkpoint Inhibition. Drugs.

[124] Gibney G.T., Weiner L.M., Atkins M.B. (2016). Predictive biomarkers for checkpoint inhibitor-based immunotherapy. Lancet Oncol..

[125] Singh M., Jadhav H.R. (2018). Targeting non-small cell lung cancer with small-molecule EGFR tyrosine kinase inhibitors. Drug Discov. Today.

[126] Luke J.J., Flaherty K.T., Ribas A., Long G.V. (2017). Targeted agents and immunotherapies: Optimizing outcomes in melanoma. Nat. Rev. Clin. Oncol..

[127] Giesen C., A O Wang H., Schapiro D., Zivanovic N., Jacobs A., Hattendorf B., Schüffler P., Grolimund D., Buhmann J.M., Brandt S. (2014). Highly multiplexed imaging of tumor tissues with subcellular resolution by mass cytometry. Nat. Methods.

[128] O’Connor M.J. (2015). Targeting the DNA Damage Response in Cancer. Mol. Cell.

[129] Coppé J.-P., Mori M., Pan B., Yau C., Wolf D.M., Ruiz-Saenz A., Brunen D., Prahallad A., Cornelissen-Steijger P., Kemper K. (2019). Mapping phospho-catalytic dependencies of therapy-resistant tumours reveals actionable vulnerabilities. Nature.

[130] Kopetz S., Desai J., Chan E., Hecht J.R., O’Dwyer P.J., Maru D., Morris V., Janku F., Dasari A., Chung W. (2015). Phase II Pilot Study of Vemurafenib in Patients with Metastatic BRAF-Mutated Colorectal Cancer. J. Clin. Oncol..

[131] Xu T., Jin J., Gregory C., Hickman J.J., Boland T. (2005). Inkjet printing of viable mammalian cells. Biomaterials.

[132] Murphy S.V., Atala A. (2014). 3D bioprinting of tissues and organs. Nat. Biotechnol..

[133] Moroni L., Burdick J.A., Highley C., Lee S.J., Morimoto Y., Takeuchi S., Yoo J.J. (2018). Biofabrication strategies for 3D in vitro models and regenerative medicine. Nat. Rev. Mater..

[134] Hospodiuk M., Dey M., Sosnoski D., Ozbolat I.T. (2017). The bioink: A comprehensive review on bioprintable materials. Biotechnol. Adv..

[135] Satpathy A., Datta P., Wu Y., Ayan B., Bayram E., Ozbolat I.T. (2018). Developments with 3D bioprinting for novel drug discovery. Expert Opin. Drug Discov..

[136] Madden L.R., Nguyen T.V., Garcia-Mojica S., Shah V., Le A.V., Peier A., Visconti R., Parker E.M., Presnell S.C., Nguyen D.G. (2018). Bioprinted 3D Primary Human Intestinal Tissues Model Aspects of Native Physiology and ADME/Tox Functions. iScience.

[137] Sambuy Y., De Angelis I., Ranaldi G., Scarino M.L., Stammati A., Zucco F. (2005). The Caco-2 cell line as a model of the intestinal barrier: Influence of cell and culture-related factors on Caco-2 cell functional characteristics. Cell Boil. Toxicol..

[138] McDonnell A.M., Dang C. (2013). Basic Review of the Cytochrome P450 System. J. Adv. Pract. Oncol..

[139] Langer E.M., Allen-Petersen B., King S.M., Kendsersky N.D., Turnidge M.A., Kuziel G.M., Riggers R., Samatham R., Amery T.S., Jacques S.L. (2019). Modeling Tumor Phenotypes In Vitro with Three-Dimensional Bioprinting. Cell Rep..

[140] Colosi C., Shin S.R., Manoharan V., Massa S., Costantini M., Barbetta A., Dokmeci M.R., Dentini M., Khademhosseini A. (2015). Microfluidic Bioprinting of Heterogeneous 3D Tissue Constructs Using Low-Viscosity Bioink. Adv. Mater..

[141] Grigoryan B., Paulsen S.J., Corbett D.C., Sazer D.W., Fortin C.L., Zaita A.J., Greenfield P.T., Calafat N.J., Gounley J., Ta A.H. (2019). Multivascular networks and functional intravascular topologies within biocompatible hydrogels. Science.

[142] Wilhelm S.M., Dumas J., Adnane L., Lynch M., Carter C.A., Schütz G., Thierauch K.-H., Zopf D. (2011). Regorafenib (BAY 73-4506): A new oral multikinase inhibitor of angiogenic, stromal and oncogenic receptor tyrosine kinases with potent preclinical antitumor activity. Int. J. Cancer.

[143] Fang Y., Eglen R.M. (2017). Three-Dimensional Cell Cultures in Drug Discovery and Development. SLAS Discov. Adv. Life Sci. R&D.

[144] Horvath P., Aulner N., Bickle M., Davies A.M., Del Nery E., Ebner D., Montoya M., Östling P., Pietiäinen V., Price L. (2016). Screening out irrelevant cell-based models of disease. Nat. Rev. Drug Discov..

[145] Prahallad A., Sun C., Huang S., Di Nicolantonio F., Salazar R., Zecchin D., Beijersbergen R.L., Bardelli A., Bernards R. (2012). Unresponsiveness of colon cancer to BRAF(V600E) inhibition through feedback activation of EGFR. Nature.

[146] Van Cutsem E., Huijberts S., Grothey A., Yaeger R., Cuyle P.-J., Elez E., Fakih M., Montagut C., Peeters M., Yoshino T. (2019). Binimetinib, Encorafenib, and Cetuximab Triplet Therapy for Patients With BRAF V600E–Mutant Metastatic Colorectal Cancer: Safety Lead-In Results From the Phase III BEACON Colorectal Cancer Study. J. Clin. Oncol..

[147] Rolfo C., Mack P.C., Scagliotti G.V., Baas P., Barlesi F., Bivona T.G., Herbst R.S., Mok T.S., Peled N., Pirker R. (2018). Liquid Biopsy for Advanced Non-Small Cell Lung Cancer (NSCLC): A Statement Paper from the IASLC. J. Thorac. Oncol..

[148] Bychkov D., Linder N., Turkki R., Nordling S., Kovanen P.E., Verrill C., Walliander M., Lundin M., Haglund C., Lundin J. (2018). Deep learning based tissue analysis predicts outcome in colorectal cancer. Sci. Rep..

[149] FOxTROT Collaborative Group (2012). Feasibility of preoperative chemotherapy for locally advanced, operable colon cancer: The pilot phase of a randomised controlled trial. Lancet Oncol..

[150] Kamps R., Brandão R., Bosch B.J.V.D., Paulussen A.D.C., Xanthoulea S., Blok M.J., Romano A. (2017). Next-Generation Sequencing in Oncology: Genetic Diagnosis, Risk Prediction and Cancer Classification. Int. J. Mol. Sci..

[151] Berger M.F., Mardis E.R. (2018). The emerging clinical relevance of genomics in cancer medicine. Nat. Rev. Clin. Oncol..

[152] Figueiras R.G., Baleato-González S., Padhani A.R., Luna-Alcalá A., Marhuenda A., Vilanova J.C., Osorio-Vázquez I., Martinez-De-Alegria A., Gomez-Caamaño A. (2018). Advanced Imaging Techniques in Evaluation of Colorectal Cancer. Radiographics.

[153] Nowak-Sliwinska P., Weiss A., Ding X., Dyson P.J., Bergh H.V.D., Griffioen A.W., Ho C.-M. (2016). Optimization of drug combinations using Feedback System Control. Nat. Protoc..

[154] Ding X., Liu W., Weiss A., Li Y., Wong I., Griffioen A.W., Bergh H.V.D., Xu H., Nowak-Sliwinska P., Ho C.-M. (2014). Discovery of a low order drug-cell response surface for applications in personalized medicine. Phys. Boil..

[155] Weiss A., Nowak-Sliwinska P. (2016). Current Trends in Multidrug Optimization: An Alley of Future Successful Treatment of Complex Disorders. SLAS Technol. Transl. Life Sci. Innov..

[156] Weiss A., Le Roux-Bourdieu M., Zoetemelk M., Ramzy G., Rausch M., Harry D., Miljkovic-Licina M., Falamaki K., Wehrle-Haller B., Meraldi P. (2019). Identification of a Synergistic Multi-Drug Combination Active in Cancer Cells via the Prevention of Spindle Pole Clustering. Cancers.

[157] Prasetyanti P.R., Van Hooff S.R., Van Herwaarden T., De Vries N., Kalloe K., Rodermond H., Van Leersum R., De Jong J.H., Franitza M., Nürnberg P. (2018). Capturing colorectal cancer inter-tumor heterogeneity in patient-derived xenograft (PDX) models. Int. J. Cancer.

